# Objective Measurement of Daytime Napping, Cognitive Dysfunction and Subjective Sleepiness in Parkinson’s Disease

**DOI:** 10.1371/journal.pone.0081233

**Published:** 2013-11-21

**Authors:** Samuel J. Bolitho, Sharon L. Naismith, Pierre Salahuddin, Zoe Terpening, Ron R. Grunstein, Simon J. G. Lewis

**Affiliations:** 1 Parkinson’s Disease Clinic, Brain and Mind Research Institute, The University of Sydney, Sydney, New South Wales, Australia; 2 Healthy Brain Ageing Clinic, Ageing Brain Centre, Brain and Mind Research Institute, University of Sydney, Sydney, New South Wales, Australia; 3 Woolcock Institute of Medical Research, University of Sydney, Sydney, New South Wales, Australia; University Medical Center Groningen UMCG, Netherlands

## Abstract

**Introduction:**

Sleep-wake disturbances and concomitant cognitive dysfunction in Parkinson’s disease (PD) contribute significantly to morbidity in patients and their carers. Subjectively reported daytime sleep disturbance is observed in over half of all patients with PD and has been linked to executive cognitive dysfunction. The current study used daytime actigraphy, a novel objective measure of napping and related this to neuropsychological performance in a sample of PD patients and healthy, age and gender-matched controls. Furthermore this study aimed to identify patients with PD who may benefit from pharmacologic and behavioural intervention to improve these symptoms.

**Methods:**

Eighty-five PD patients and 21 healthy, age-matched controls completed 14 days of wrist actigraphy within two weeks of neuropsychological testing. Objective napping measures were derived from actigraphy using a standardised protocol and subjective daytime sleepiness was recorded by the previously validated Epworth Sleepiness Scale.

**Results:**

Patients with PD had a 225% increase in the mean nap time per day (minutes) as recorded by actigraphy compared to age matched controls (39.2 ± 35.2 vs. 11.5 ± 11.0 minutes respectively, p < 0.001). Significantly, differences in napping duration between patients, as recorded by actigraphy were not distinguished by their ratings on the subjective measurement of excessive daytime sleepiness. Finally, those patients with excessive daytime napping showed greater cognitive deficits in the domains of attention, semantic verbal fluency and processing speed.

**Conclusion:**

This study confirms increased levels of napping in PD, a finding that is concordant with subjective reports. However, subjective self-report measures of excessive daytime sleepiness do not robustly identify excessive napping in PD. Fronto-subcortical cognitive dysfunction was observed in those patients who napped excessively. Furthermore, this study suggests that daytime actigraphy, a non-invasive and inexpensive objective measure of daytime sleep, can identify patients with PD who may benefit from pharmacologic and behavioural interventions to improve these symptoms.

## Introduction

Sleep-wake disturbance is gaining increased attention in Parkinson’s disease (PD). Such problems are observed in over two thirds of patients [1] manifesting with a range of sleep symptoms [2]. In addition to nocturnal sleep disturbance, daytime sleep disturbance is defined as encompassing both excessive daytime sleepiness (EDS) and excessive daytime napping, which are frequently observed in patients with PD [3,4]. Whilst EDS and excessive daytime napping are separate constructs within sleep medicine, the distinction of these phenomena within PD cohorts remains less clear. Previous studies have considered these symptoms both as separate entities [5,6] and also as being measures along the same continuum within PD cohorts [3,4,7-9] . It is possible that the overlap of these phenomena in part represents a common neural and chemical mechanism involving the reticular activating system of the brainstem, basal forebrain and hypothalamus through activation of the homeostatic sleep system [10,11]. Thus it is difficult to make a clear distinction between excessive daytime sleepiness and excessive daytime napping in PD. 

In addition to daytime sleep disturbance, fatigue is frequently observed within PD cohorts. It is important to distinguish fatigue, an overwhelming lack of energy [12] from excessive daytime sleepiness and excessive daytime napping, which exist with impairment of normal arousal mechanisms [12]. Previous studies in PD have identified a link between the self-reported tendency to doze or nap in contrast to fatigue , measured with the Epworth Sleepiness Scale [13] and cognitive deficits [14-16]. Non-motor symptomatology in PD, including cognitive dysfunction and daytime somnolence contribute significantly to poor quality of life for patients and their carers [17]. Cognitive deficits are an independent predictor of admission to a nursing home, conferring a negative impact of burden of health to the community [18]. However these relationships in daytime somnolence have yet to be investigated using an objective measure. 

The development of daytime somnolence in PD has been associated with increasing age, disease duration, disease progression, postural instability, depression and the use of dopamine agonists [8,19,20]. However, much like the emergence of idiopathic REM sleep behavior disorder (RBD) in later life [21], daytime sleep disturbance can also represent a pre-motor feature heralding the development of PD [3,4,14]. Daytime sleep disturbance has been linked to executive dysfunction in PD [16] and impairments in frontostriatal neural circuitry have been implicated in the reduced arousal, attentional modulation and general working memory seen in PD [16,22,23]. These observations may imply a link between excessive napping and reduced cognition in PD [14-16]. 

The pattern of neuronal loss and neurotransmitter deficits giving rise to daytime sleep disturbance in PD are not well understood but dopaminergic and non-dopaminergic pathology across the brainstem, basal forebrain, hypothalamus and frontostriatal pathways have been suggested [3,7,10,14]. Such pathology may impair wake promoting structures [11,24,25] and/or possibly disrupt the proposed sleep homeostat [25]. The control of this sleep homeostat is not well defined, but some have suggested it operates via the accumulation of activity dependent metabolites that promote sleep throughout the day (including adenosine, gamma amino butyric acid (GABA), prostaglandin D2 (PGD2), interlukin–1A (Il-1A) and tumour necrosis factor-alpha (TNFα)) [10].

Previous studies in non-PD samples have shown that older individuals with EDS are more likely to nap during the day [26,27]. More extensive work has been conducted on napping in healthy, older adults where it appears to be associated with increased morbidity [28] and mortality [29]. In these older cohorts increased napping has been associated with decreased global cognition [26,30-32] and in particular deficits in executive function [31]. Interestingly, studies that have utilised ‘prescribed’ napping to restore the effects of sleep deprivation in healthy cohorts, have demonstrated improved executive performance on tasks such as reaction time and symbol digit substitution [33-35]. These combined observations highlight the possibility that the increased frequency of napping seen in older adults and patients with PD might represent a compensatory neurobiological strategy to a primary neuropathological insult (rather than playing a causative role in cognitive deficits).

Studies in non-PD cohorts have successfully utilised daytime actigraphy as a non-invasive measure of daytime napping [32,36-38]. Furthermore, the use of actigraphy to explore sleep disturbance has been well validated in nocturnal sleep disturbance in PD [39,40]. The current study sought to compare objective and subjective measurement of daytime sleep disturbance in a sample of PD patients and a group of age matched healthy controls that had all undergone neuropsychological testing. 

This is the first study to compare the ESS, a widely used self-report questionnaire that rates the probability of napping, with an objective measure of napping. We hypothesised that the duration of daytime napping would be greater in PD patients as compared to controls and that excessive napping would be associated with impaired cognitive performance, specifically within domains mediated by fronto-subcortical circuitry. Furthermore we propose that as the ESS rates the probability of napping in several situations, patients with excessive daytime sleepiness as determined by the ESS, should also exhibit greater levels of napping as identified objectively by actigraphy Finally, we suggest that the objective measurement of daytime napping will more accurately identify those patients who may benefit from pharmacologic and behavioural interventions to improve these symptoms.

## Methods

### Ethics statement

Permission for the study was obtained from the University of Sydney Human Research Ethics Committee (HREC 02-2008/11105). All patients gave written informed consent.

### Participants

Eighty five patients and 21 age matched healthy controls were recruited from the Brain & Mind Research Institute (BMRI) PD Research Clinic, University of Sydney. All participants with a known or suspected diagnosis of obstructive sleep apnea were excluded, including any participant who had previously had CPAP prescribed or who had greater than mild OSA on a diagnostic sleep study [16]. Patients were then asked three screening questions to identify snoring, nocturnal snorting or gasping or a history of nocturnal apneas and were excluded if these were present. No patients were demented as assessed by the Movement Disorders Society criteria [41] and participants with a diagnosis of major depression were excluded. Five patients were unmedicated, thirty patients were on levodopa monotherapy, six were on dopamine agonist monotherapy, forty were on levodopa plus an adjuvant agent (e.g. dopamine agonist, COMT inhibitor, MAO inhibitor), three were on a dopamine agonist plus amantadine and one was on a dopamine agonist plus Rasagiline. Thirteen patients with PD were taking medications to aid sleep. Twelve of these were taking a benzodiazepine and one was taking melatonin. None of the controls were taking sleeping medications. Five patients had deep brain stimulators in situ. 

### Clinical assessment

All neurological and neuropsychological assessments were conducted within one session to confirm study eligibility. Patients were assessed in their ‘on’ state and levodopa dose equivalents were calculated for dopaminergic medication [42]. Disease stage was rated on the Hoehn and Yahr (H&Y) scale [43], disease duration was calculated from time since disease diagnosis, and depressive symptoms were self-rated using the Beck Depression Inventory–II (BDI-II, scores of 0-13 indicative of minimal depressive symptoms) [44]. 

Neuropsychological functioning was assessed within the PD cohort using standardised tests and appropriate normative data (with corrections for age and level of education). These variables were included in the healthy control group for descriptive purposes only. Language generativity was assessed with semantic verbal fluency via the Controlled Oral Word Associated Test (COWAT animals; z-score) [15,45]. Set-shifting was measured using the Trailmaking Test, Part B (TMT-B; z-score) [46,47]. Processing speed was assessed using the choice reaction time test from the Cambridge Neuropsychological Test Automated Battery (CANTAB; z-score) [16,48]. The Mini Mental State Examination (MMSE) [49] was administered for reporting purposes. Similarly the ability to retain learned verbal memory was assessed using the Logical Memory (percentage retention) subtest from the Wechsler Memory Scale - III [50] and working memory, assessed using the Digit Span backwards subtest of the Wechsler Adult Intelligence Scale – III (raw score) [51] were included for reporting purposes.

### Actigraphic assessment

The use of actigraphy to assess daytime sleep has been validated previously in healthy subjects in both the laboratory and community setting [37,38] and the measurement of daytime sleep-wake disturbance in this study was conducted according to previously established protocols [46,52]. Following clinical assessment, participants were required to wear a wrist actiwatch (Minimitter Actiwatch Spectrum) on the wrist less affected by tremor every day for fourteen days. Actigraphy rest intervals were calculated using Actiware 5.0 software (Minimitter-Respironics Inc, Bend, Oregon) in conjunction with manual scoring by an experienced sleep technician. An episode of daytime sleep was defined as resting with no movement on actigraphy during the day for a minimum duration of thirty minutes. The primary measure of daytime sleep was the nap time per day (minutes) which was calculated by summing all napping each day and averaging this over the 14 day measurement period. The number of nap bouts per day were also reported. Total nocturnal sleep time (TST), wake after sleep onset (WASO) and sleep efficiency ((TST – WASO)/TST) were also derived from the actiwatch as per previously established protocols [39,46]. Patients were defined as exhibiting ‘excessive daytime napping’ if their nap time per day was greater than a threshold derived from the control data. This threshold was defined as the average nap time per day (duration) + 1.5 standard deviations (minutes).

### Subjective Assessment of Daytime Sleep Disturbance

Patients were asked to complete the Epworth Sleepiness Scale (ESS) (score ≥ to 10 indicative of a high probability of daytime sleep) [13], within two weeks of completing the actigraphy.

### Statistical analysis

Statistical analysis was conducted on PASW Statistics Version 20 for Windows. Age was compared between the groups using a t-test. Gender was compared using a chi-square test. Subsequent variables violated assumptions of normality and non-parametric Mann-Whitney U test were used for these comparisons. All tests were two-tailed with an α value of 0.05. The three cognitive variables were compared between groups utilising a Bonferroni correction for multiple comparisons.

## Results

### Patients vs. Controls

As shown in Table 1, there was no significant difference in age or gender between the PD group and control groups. The groups were not different with regard to global cognition (i.e. MMSE). However, the patient group had higher ESS scores (p=0.001). As measured by the BDI-II, depressive symptoms were significantly higher in the PD group by an average of five points (p<0.001). However, the average BDI-II in the PD group was only 9.2 (SD 6.7), suggestive of only minimal depression. 

**Table 1:** Descriptive, neurologic, sleep and cognitive data for patients and controls.

	Controls	Parkinson's Disease	Statistic	p-value
	Mean ± SD (n = 21)	Mean ± SD (n = 85)		
Age (years)	63.4 ± 9.5	64.8 ± 7.4	t = -0.6	0.537
Sex, Male: Female	12:9	53:32	χ^2^ = 0.2	0.661
Hoehn and Yahr		2.0 ± 0.7		
Disease duration (years)		5.9 ± 5.2		
Levodopa dose equivalent (mg)		641.9 ± 466.3		
Participants taking sleeping tablets	0	13		
Participants with DBS in situ		5		
Average nap time per day (min)	11.5 ± 11.0	39.2 ± 35.2	U = 345.0	< 0.001
Median average nap time per day (min) (IQR)	7.7 ± 16.7	26.5 ± 34.6	U = 345.0	< 0.001
Average naps per day	0.2 ± 0.3	0.6 ± 0.5	U = 375.0	< 0.001
Total nocturnal sleep time (min)	438.0 ± 39.2	453.0 ± 67.1	U = 762.5	0.303
Sleep efficiency (%)	91.1 ± 3.0	90.2 ± 4.7	U = 767.5	0.602
Wake after sleep onset (min)	35.3 ± 8.4	34.3 ± 12.0	U = 713.0	0.329
Epworth Sleepiness Scale	4.6 ± 3.6	8.1 ± 4.3	U = 483.5	0.001
Number of participants with ESS ≥ 10	3	33	χ^2^ = 4.521	0.033
Beck Depression Inventory-II	3.1 ± 3.9	9.2 ± 6.7	U = 338.0	< 0.001
Mini-Mental State Examination	28.4 ± 1.6	28.4 ± 1.8	U = 851.0	0.732
Logical Memory retention (% retention)	10.9 ± 3.3	11.0 ± 3.4	U = 890.0	0.984
Digit span backwards raw score	7.1 ± 2.3	6.8 ± 1.8	U = 885.0	0.952
Verbal Fluency animals z-score	0.5 ± 1.6	0.1 ± 1.3	U = 827.5	0.606
Trailmaking Test, Part B z-score	-0.1 ± 1.6	-0.9 ± 1.8	U = 627.5	0.016
Choice reaction time z-score	-0.02 ± 1.2	-0.1 ± 1.4	U = 884.0	0.946

IQR, interquartile range.

**Figure 1:**
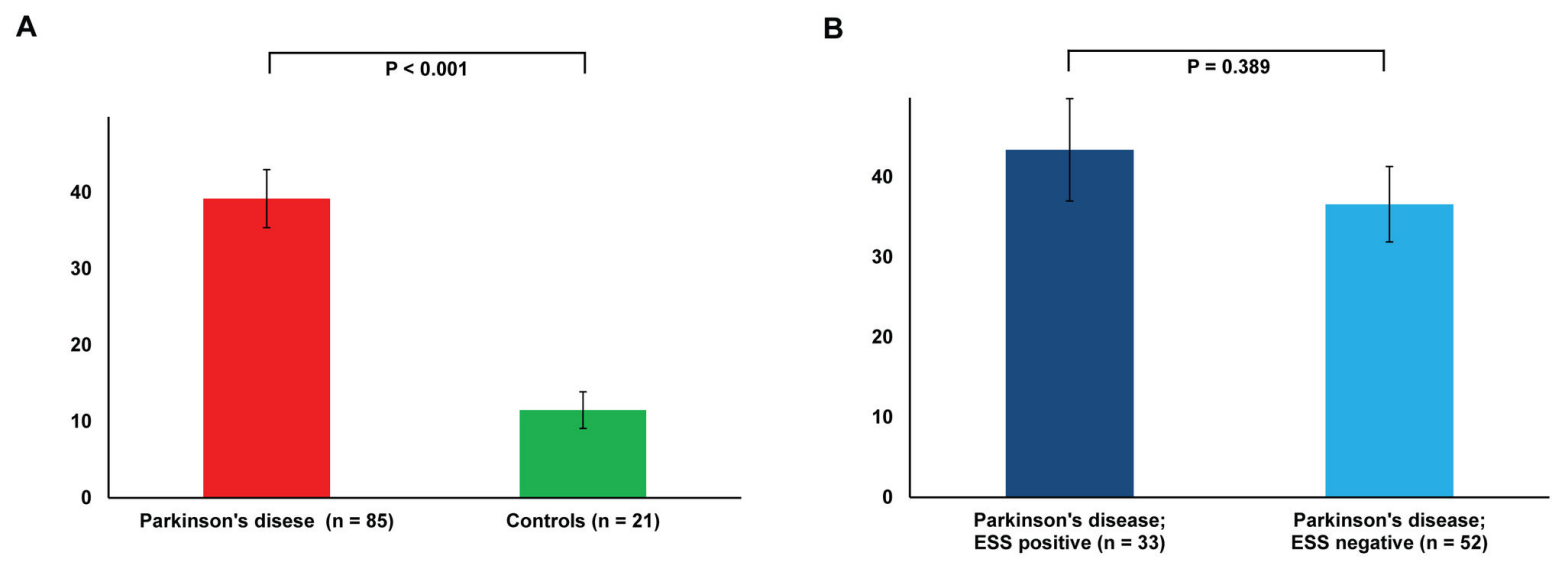
Average nap time per day (minutes). A chart depicting the average nap time per day (± standard error) calculated by summing the daytime napping periods identified by actigraphy and averaging this over the 14 day measurement period. Panel A - Parkinson’s disease vs. Controls. Panel B - Parkinson’s disease patients divided into those who are Epworth Sleepiness Scale positive (score ≥ to 10 indicative of sleepiness) vs. Parkinson’s Disease patients who are Epworth Sleepiness Scale negative.

Napping data shown in Figure 1 reports that patients in this study had a 225% increase in the mean nap time per day compared to age matched controls (39.2 ± 35.2 vs. 11.5 ±11.0 minutes respectively, p < 0.001). Similarly there was a 244% increase in median nap time per day in the (p=0.003) and significantly more nap bouts per day when comparing the patient group to controls (0.6 ± 0.5 vs. 0.2 ± 0.3 respectively, p < 0.001). To ensure that the increased napping noted in the PD group was not due to sleep deprivation, the average total nocturnal sleep time, derived from actigraphy over the fourteen day sampling period, was compared to controls. There was no difference in this measure between the two groups to suggest the patients with PD had a sleep debt (p=0.303). Furthermore, there was no difference in sleep efficiency (p = 0.602) or wake after sleep onset (p = 0.329) comparing the PD group to controls. 

As mood disturbance has been linked previously to daytime somnolence [53], an analysis of co-variance (ANCOVA) was performed to assess the contribution of mood disturbance (BDI-II) to the measure of nap time per day between PD and control groups. This result affirmed the increased nap time per day seen in the PD group compared to controls and remained significantly increased when mood disturbance (BDI-II) was used as a covariate (F = 9.6, p =0.003). This result was further corroborated by the finding that BDI-II did not correlate with nap time per day in the PD group (Spearman rho = 0.095, p = 0.390). 

### Patients with Excessive Daytime Napping vs. Patients without Excessive Daytime Napping

**Figure 2:**
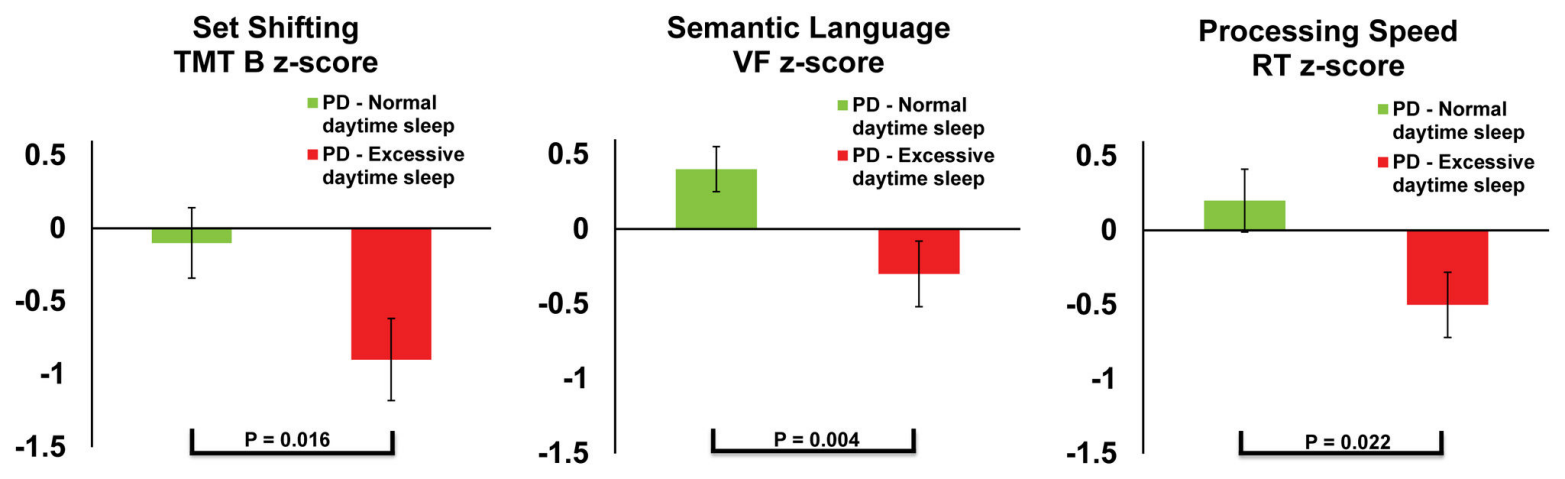
Cognitive performance of excessive nappers within the Parkinson’s disease cohort. A chart comparing the cognitive performance (mean ± standard error) of patients with Parkinson’s disease (PD) divided into those with excessive daytime napping vs. those with normal daytime napping. Set shifting was measured with the Trailmaking task part B (TMT B; z score). Semantic verbal fluency (VF) was tested via the Controlled Oral Word Associated Test (COWAT animals; z score) and processing speed was measured with the choice reaction time (RT) test from the Cambridge Neuropsychological Test Automated Battery (CANTAB; z score).

Table 2 shows the comparison of patients with (n=41) and without (n=44) excessive daytime napping. These groups showed no differences in their disease duration, disease stage or levodopa dose equivalent. Furthermore, patients on sleeping tablets were not over represented in either the excessive or normal napping group (χ^2^ = 0.194, p = 0.660). Similarly, global cognition (MMSE), mood disturbance (BDI-II), retention of learned verbal memory (Logical Memory percentage retention), and working memory (Digit Span backwards) were not different between the groups. As age was noted to be different between the two sub-groups of PD patients, age-adjusted normative z-scores were used. As shown in Figure 2, patients who exhibited excessive daytime napping had significantly poorer mental flexibility and set-shifting (TMT-B z-score, p=0.016), and semantic verbal fluency (COWAT animals z-score, p=0.004). Though the processing speed was also slower in those with excessive napping this did not meet the correction for multiple comparisons and represents a trend (choice reaction time z-score, p=0.022). Within the PD cohort, there was no evidence of a deficit in nocturnal sleep (total nocturnal sleep time p = 0.356), sleep efficiency (p = 0.800) or wake after sleep onset (p = 0.544), that could explain the excessive daytime napping and cognitive deficit seen in these results. Furthermore, the patients with DBS were not over represented in either excessive or normal nappers (χ^2^ = 0.294, p = 0.587).

**Table 2:** Descriptive, neurologic, sleep and cognitive data in Parkinson’s disease patients: excessive vs. normal daytime sleep.

	Normal daytime sleep	Excessive daytime sleep	Statistic	P -value
	Mean ± SD (n=44)	Mean ± SD (n = 41)		
Age (years)	62.4 ± 7.1	67.3 ± 7.0	t = -3.3	0.001
Hoehn and Yahr	2.0 ± 0.6	2.1 ± 0.8	U = 828.5	0.485
Disease duration (years)	5.8 ± 4.7	5.8 ± 5.6	U = 825.5	0.501
Levodopa dose equivalent (mg)	675.7 ± 516.2	605.6 ± 409.3	U = 855.0	0.679
Participants taking sleeping tablets	5	7	χ^2^ = 0.194	0.660
Participants with DBS in situ	2	3	χ^2^ = 0.294	0.587
Average nap time per day (min)	14.9 ± 7.6	65.3 ± 34.6	U = 0.0	<0.001
Average naps per day	0.3 ± 0.2	1.0 ± 0.5	U = 56.0	<0.001
Total nocturnal sleep time (min)	446.0 ± 55.5	460.7 ± 77.1	U = 797.0	0.356
Sleep efficiency (%)	90.0 ± 5.1	91.0 ± 4.2	U = 831.5	0.800
Wake after sleep onset (min)	35.7 ± 13.3	32.9 ± 10.4	U = 793.5	0.544
Epworth Sleepiness Scale	7.8 ± 4.7	8.6 ± 3.9	U = 760.0	0.210
Beck depression inventory-II	8.8 ± 7.3	9.6 ± 6.1	U = 780.5	0.365
Mini-Mental State Examination	28.5 ± 1.8	28.4 ± 1.8	U = 877.0	0.819
Logical Memory retention (% retention)	11.0 ± 3.6	11.0 ± 3.4	U = 900.5	0.989
Digit span backwards raw score	6.9 ± 1.8	6.8 ± 1.9	U = 868.0	0.761
Verbal Fluency animals z-score	0.4 ± 1.0	-0.3 ± 1.4	U = 576.0	0.004
Trailmaking Test, Part B z-score	-0.1 ± 1.6	-0.9 ± 1.8	U = 627.5	0.016
Choice reaction time z-score	0.2 ± 1.4	-0.5 ± 1.4	U = 641.0	0.022

**Figure 3:**
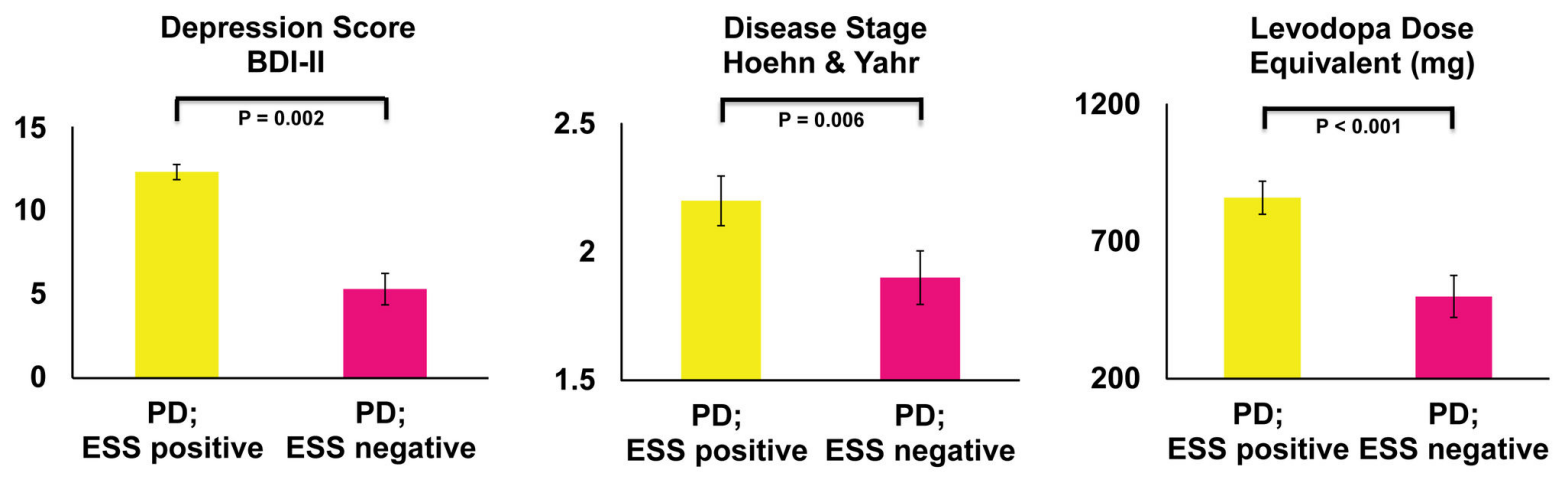
Mood and disease specific variables within the Parkinson’s disease cohort based on subjective sleepiness scores. A chart that reports depression scores, disease stage and Levodopa dose equivalents (mean ± standard error) when patients with Parkinson’s Disease are divided into those who are Epworth Sleepiness Scale positive (score ≥ to 10 indicative of a positive tendency to sleep during the day) vs. those who are Epworth Sleepiness Scale negative.

By contrast, differences in napping duration between patients with and without excessive daytime napping as recorded by actigraphy were not distinguished by their ratings on the ESS. Table 3 shows results comparing patients with PD, divided into those with a tendency to nap during the day based on an ESS ≥ 10. Those who were positive on the ESS also had poorer set-shifting (TMT-B z-score p = 0.005) and a trend to reduced processing speed when adjusting for multiple comparisons (choice reaction time z-score, p = 0.047). Although not the primary focus of this study, those who were positive on the ESS also had a trend towards poorer working memory (digit span backwards raw score, p = 0.020). However, unlike when the group was divided by excessive napping identified with actigraphy, Figure 3 reports that patients identified to be positive on the ESS, had significantly greater mood deficit (p = 0.006), disease stage (p = 0.006) and levodopa dose equivalent (p < 0.001). 

**Table 3:** Parkinson’s disease patients: Epworth sleepiness scale (ESS) positive v Epworth sleepiness scale (ESS) negative.

	ESS positive	ESS Negative	Statistic	P -value
	Mean ± SD (n=33)	Mean ± SD (n = 52)		
Age (years)	64.5 ± 6.6	65.0 ± 8.0	t = 0.3	0.783
Hoehn and Yahr	2.2 ± 0.7	1.9 ± 0.6	U = 573.0	0.006
Disease duration (years)	6.9 ± 5.1	5.3 ± 5.2	U = 667.0	0.085
Levodopa dose equivalent (mg)	858.9 ± 434.4	500.0 ± 439.1	U = 458.0	< 0.001
Participants taking sleeping tablets	5	8	χ^2^ = 0.001	0.977
Participants with DBS in situ	1	4	χ^2^ = 0.793	0.373
Average nap time per day (min)	43.4 ± 37.0	36.6 ± 34.1	U = 762.5	0.389
Average naps per day	0.6 ± 0.5	0.7 ± 0.5	U = 783.5	0.500
Epworth Sleepiness Scale	12.5 ± 2.6	5.3 ± 2.6	U = 0.0	< 0.001
Beck depression inventory-II	12.3 ± 7.5	7.3 ± 5.4	U = 508.5	0.002
Mini-Mental State Examination	28.2 ± 1.9	28.6 ± 1.8	U = 772.0	0.418
Logical Memory retention (% retention)	11.0 ± 3.6	11.0 ± 3.4	U = 900.5	0.358
Digit span backwards raw score	6.2 ± 1.9	7.3 ± 1.8	U = 868.0	0.020
Verbal Fluency animals z-score	-0.04 ± 1.0	0.2 ± 1.4	U = 768.0	0.417
Trailmaking Test, Part B z-score	-1.2 ± 2.0	-0.06 ± 1.4	U = 544.0	0.005
Choice reaction time z-score	-0.6 ± 1.6	0.2 ± 1.2	U = 638.0	0.047

## Discussion

This study is the first to use actigraphy, a previously validated objective measure of daytime sleep, to record the duration and correlates of excessive daytime napping in PD. Patients with PD, reported significantly greater number of nap bouts as well as time spent napping in the day, compared to healthy age matched controls. Patients with PD who exhibited excessive napping through the day had poorer performance on neuropsychological tests probing fronto-subcortical functions including set-shifting, semantic verbal fluency and processing speed. This result is in keeping with previous findings evaluating excessive daytime somnolence [16]. However, other studies have suggested prescribed napping can improve cognition. This paradox may imply that excessive napping is a compensatory process to preexisting cognitive deficit. Alternatively these results may imply the neural and chemical processes that bring about excessive or uncontrolled napping, rather than intended or prescribed napping are linked to the cognitive dysfunction seen in this study. 

These results were not accounted for by age, mood disturbance, dementia, disease duration, disease stage or levodopa dose equivalent. As patients with obstructive apnea were excluded from the trial, this common cause of daytime sleepiness could not explain the excessive daytime napping seen in the PD group. Furthermore there was no evidence of sleep deprivation or poor sleep quality in patients compared to controls that may be an alternative cause of the increased daytime napping seen. Of note there was no difference in total sleep time, sleep efficiency or wake after sleep onset between patients with PD and controls. This finding is consistent with previous studies in this area although mixed results have been reported, which may reflect issues of sample size (for review see 54). Within the PD group, those with excessive daytime napping did not exhibit less nocturnal sleep time, sleep efficiency or wake after sleep onset time. 

Within this study, the ESS could not discriminate between patients with and without excessive daytime napping that was identified with actigraphy. This result may reflect the fact that EDS and excessive daytime napping are actually separate constructs. However, several earlier studies have suggested an overlap between EDS and excessive daytime napping exists within PD cohorts [3,4,7-9]. Thus the results presented here suggest that the ESS may not be an ideal measure of all elements of daytime sleep disturbance within PD patients. 

This study suggests that interventions aimed at reducing daytime sleep disturbance in PD may have additional benefits on cognition. Previously, the psychomotor stimulant modafinil has been investigated for the treatment of EDS in PD with mixed results [55-57]. Modafinil is believed to promote wakefulness by inhibiting a dopamine re-uptake and may also affect noradrenergic reuptake. Other wake promoting agents such as sodium oxybate and caffeine may act to reduce the effects of activity dependent metabolites that promote the global dampening of wake promoting structures and corresponding neurotransmitters release [58] have also been trialed in PD [59]. However, these studies did not identify patients with daytime sleep disturbance using an objective measure such as actigraphy. Rather, they used the ESS, which in this study did not identify excessive daytime napping. Therefore, future studies utilising this objective measurement may identify a target cohort of PD in which to better assess the efficacy of pharmacologic and behavioural interventions for these symptoms.

A similar pattern of cognitive dysfunction was also noted when dividing the group into those with daytime sleep disturbance on either the ESS or actigraphy despite the ESS positive group not identifying higher amounts of napping. This result implies that both measurements are tapping into the same subset of patients with PD. However, patients who were positive on the ESS also had higher levels of depression and more advanced disease both of which are known to be independent predictors of cognitive dysfunction. These confounds were not identified when dividing the group based on actigraphy implying that the ESS may be affected by other non-sleep related symptoms. Establishing the reason why the ESS was not able to identify excessive napping was beyond the scope of this study. However, it may be that the ESS is tapping into the akinetic rigid phenotype of PD within which EDS, excessive daytime napping, depression and cognitive dysfunctions exist in varying combinations. Further studies are needed to confirm if scores on the ESS confounded by concomitant problems associated with the sleep disturbance in PD such as depression and more advanced disease. 

Results from this study suggest a common pathology linking excessive daytime napping and specific domains of cognitive function in PD. However, putative mechanisms explaining this link have not been elucidated. Previous studies have suggested that daytime sleep disturbance might arise from damage to wake promoting structures in the brain stem, basal forebrain and hypothalamus or corresponding deficit in wake promoting neurotransmitters. It is difficult to infer that the executive cognitive deficit seen in this study may result directly from these changes. However, pathology across thalamocortical, hypothalamocortico and basalocorticoal circuitry could explain the cognitive dysfunction observed. Although there was no evidence of sleep debt that could explain the link between cognition dysfunction and excessive napping seen in patients with PD, this study was not able to exclude poorly consolidated sleep as a cause for these results. Further studies using power spectral analysis of polysomnography will help determine if the cognitive deficit seen in these results correlates with a specific deficit of sleep microarchitecture.

In animal models, adenosine and other activity dependent metabolites have been shown to facilitate both global dampening of cortical activity in addition to directly inhibiting wake promoting structures such as the cholinergic pedunculopontine nucleus [11]. Given metabolic byproducts could be increased in an oxidative stress model of PD [60], it is possible that this neurochemical process may be contributing to the link between excessive daytime napping and cognition observed in this study. Calmodulin dependent kinase II has been shown to play a causal role in cognitive and motor deficits in animal models of PD. This chemical is critical to establishing NREM sleep architecture and is also linked to synaptic plasticity and learning [61]. Thus, alterations in calmodulin dependent kinase II levels may provide a novel explanation of these results representing a common pathological mechanism between daytime sleep disturbance and cognitive deficit in PD [62-64]. 

The difference in means for these cognitive variables across the two groups ranged from 0.7 to 0.8 standard deviations and may be interpreted as modest. However, this study was not powered sufficiently to determine the effect size of the impaired cognition linked to excessive napping and this represents a limitation of the study. Further studies measuring excessive napping measured prospectively are needed to determine this effect size and the impact the cognitive deficit has on functional status and quality of life.

The ESS does not, by its intended design have a definitive time scale over which the daytime sleep disturbance is assessed. Instead it asks participants to rate their probability of napping “in recent times”. This represents a limitation when comparing the ESS with actigraphy. To minimise this limitation the ESS was administered within two weeks of completing the actigraphy. Reassuringly, studies in non PD cohorts have shown the ESS to have minimal variability over periods longer than this window [65,66]. Furthermore, although actigraphy is a validated measure of sleep, it cannot confirm the cortical EEG correlates of sleep architecture. The lack of polysomnography also represents a limitation in this study. Finally, it is possible that actigraphy may under or over classify daytime sleep based on extra movements or a lack of movement respectively. Further studies characterising this limitation in PD cohorts are needed. Studies using daytime sleep diaries rather than the ESS may also provide better subjective measurement of daytime napping for future comparison with actigraphy.

In summary, using an objective measurement this study has confirmed that patients with PD exhibit excessive napping compared to healthy age matched controls. Conflicting results between self-report questionnaires (namely the widely used ESS) and wrist actigraphy confirm the need for more objective measurement of daytime sleep-wake disturbance. Further, those patients with PD who nap excessively during the day have greater cognitive deficits in the domains of attention, semantic verbal fluency and processing speed. These results highlight a possible interrelationship between sleep and cognitive circuitry in PD that may represent common pathology. Further studies are now needed to evaluate the effect of prescribed napping, targeted at those with excessive daytime napping. Furthermore, given the potential for pharmacological and behavioural interventions to reduce excessive napping, trials are needed to investigate if these treatments can improve focal cognitive deficits in PD. This would have far reaching benefit to the quality of life of patients and their carers, in addition to reducing the burden of illness in the community.
